# The strip-shaped deep white matter hyperintensities may be related to neurodegeneration: A study based on diffusion tensor imaging

**DOI:** 10.1371/journal.pone.0354301

**Published:** 2026-07-20

**Authors:** Xuexia Sheng, Long Xu, Donghua Xu, Daihai Yuan, Wenchao Xie, Jiyuan Ge, Yizhi Zhao, Chaoyue Yang, Zhigang Min

**Affiliations:** 1 Department of Radiology, The Affiliated Yixing Hospital of Jiangsu University, Yixing, Jiangsu, China; 2 Department of Neurology, The Affiliated Yixing Hospital of Jiangsu University, Yixing, Jiangsu, China; University of Thessaly Faculty of Medicine: Panepistemio Thessalias Tmema Iatrikes, GREECE

## Abstract

**Purpose:**

This study investigated the properties of strip-shaped white matter hyperintensities (WMHs) by examining the relationship between their principal axes and maximum diffusion direction, and comparing their diffusion tensor metrics with punctate and early confluent WMHs to explore their pathological features and potential link to chronic axonal injury.

**Methods:**

Eighty-seven participants with isolated WMHs were included and received diffusion tensor imaging (DTI) using a 3.0-T MR scanner. Lesions were classified by elongation (strip-shaped: ≥ 2; punctate: < 2). We recorded the maximum diffusion direction of punctate, strip-shaped, and early confluent WMHs and evaluated their consistency with the principal axial direction. Additionally, the diffusion tensor metrics of strip-shaped WMHs were measured and compared with those of punctate and early confluent WMHs.

**Results:**

Among 996 WMHs, 57.5% of strip-shaped lesions showed principal axis alignment with the maximum diffusion direction, significantly higher than punctate (44.7%) and early confluent (42.1%) lesions (P < 0.05). Mixed-effects models showed significant lesion type effects on FA, MD, AD, and RD (all P < 0.05). Post-hoc comparisons revealed that stripe lesions had higher FA but lower MD and RD than confluence lesions (all P < 0.05), with no significant differences between stripe and punctate groups for MD or RD. No AD differences were found among groups. Fiber tracking confirmed alignment in most strip-shaped lesions.

**Conclusion:**

Strip-shaped WMHs exhibit unique diffusion metrics and fiber alignment, suggesting chronic axonal injury due to neurodegeneration. These findings underscore the importance of morphological and microstructural analysis in understanding WMH heterogeneity and clinical significance.

## Introduction

White matter hyperintensities (WMHs), commonly observed on magnetic resonance imaging (MRI), are presumed to be a manifestation of cerebral small vessel disease. These lesions appear as areas of high signal intensity on T2-weighted or FLAIR MRI sequences [1]. The Fazekas visual scoring system [2,3] classified deep WMHs into three grades: punctate (grade 1), beginning confluent (grade 2), and large confluent (grade 3) foci. While grades 2 and 3 have been extensively studied due to their association with cognitive impairment [4,5], the correlation between WMH grade and cognitive performance remains modest [6]. Postmortem and MRI studies reveal histopathological heterogeneity in WMHs, ranging from mild matrix disentanglement to varying degrees of myelin and axonal loss [7]. This variability may partly explain the limited imaging-clinical symptom correlation [8]. Consequently, identifying distinct WMH subtypes with differing etiologies represents a promising research direction [9].

Morphological analysis of WMHs may provide further insights into their etiology and prognosis [10,11]. Specific WMH shapes have been linked to long-term dementia risk in older adults [10], as well as mortality and ischemic stroke in patients with arterial disease [12]. Additionally, WMH morphology has been associated with cognitive function [13]. Strip-shaped WMHs are commonly observed but have not been thoroughly investigated. Notably, they can present as punctate lesions when imaged perpendicular to the scanning plane. De Bresser et al.[14] reported that WMH eccentricity (defined as the ratio of the maximum to minimum diameter) was higher in patients with type 2 diabetes than in controls, suggesting that increased eccentricity may serve as a quantifiable marker of punctate deep WMHs progressing toward ischemic changes-a potential indicator of early confluent WMHs. Loos et al.[15] further demonstrated that WMHs can arise secondary to lacunar infarcts and may be associated with Wallerian degeneration, a proposed contributor to WMH formation [16]. However, it remains unclear whether strip-shaped WMHs represent punctate lesions, early-stage confluent lesions, or neural degeneration. Aside from Wallerian degeneration secondary to lacunar infarcts, several other mechanisms may contribute to strip-shaped WMH formation. These include chronic ischemic injury along penetrating arteriolar territories [17], perivenous edema from venous collagenosis or outflow obstruction [18], and degenerative changes associated with upstream cortical pathology [19]. The mixed etiology of punctate lesions demonstrated by lesion probability mapping further suggests that strip-shaped WMHs may similarly reflect heterogeneous pathological processes, underscoring the need for imaging methods such as diffusion tensor imaging(DTI) to characterize their underlying microstructure.

DTI is an MRI technique capable of detecting microstructural white matter changes. Prior studies have shown that demyelination elevates radial diffusivity (RD), whereas axonal injury alters axial diffusivity (AD) [20]. In our previous work, DTI parameter comparisons across paraventricular WMH grades supported the demyelinating nature of these lesions [21]. If strip-shaped lesions are linked to Wallerian degeneration, their long-axis orientation should align with the white matter’s principal diffusion direction, and AD values may reflect axonal injury. In this study, we employed DTI to investigate the relationship between the principal axes of strip-shaped WMHs and the maximum diffusion direction of white matter, aiming to elucidate their underlying properties. Additionally, we compared diffusion parameters among strip-shaped, punctate, and early confluent lesions to characterize their potential pathological differences.

## Materials and methods

### Participants

A retrospective analysis was conducted using data from local community residents who underwent physical examinations at our institution. This study received written approval from the Ethics Committee of the Affiliated Yixing Hospital of Jiangsu University. Due to the retrospective and anonymized nature of the data, informed consent was waived. The relevant patient data were extracted from the hospital’s central digital archive, with the final research dataset created and accessed on 20 December 2019. To ensure patient confidentiality, all direct identifiers (name, national ID number, contact information) were removed by the hospital’s data custodians before the dataset was released to the research team. Thus, the analysis was performed on a fully anonymized dataset. Cases with isolated punctate, strip-shaped, and early confluent lesions in deep white matter on the FLAIR images were included. Exclusion criteria were: 1) a history of infarction, tumor, encephalitis, or other diseases affecting white matter; 2) blurred images due to severe motion artifacts; 3) lesions confluent with periventricular WMHs due to their distinct properties. A total of 87 cases were included, comprising 46 males and 41 females, with an average age of 66.9 years (range: 41–85 years).

### DTI and post-processing

Diffusion-weighted images (DWIs) were acquired using a 3.0-T MR scanner (Philips Achieva, Best, the Netherlands) with a 16-channel head-neck phased array coil. Imaging parameters included: slice thickness of 2 mm with no gaps, repetition time of 8000 ms, echo time of 87 ms, field-of-view of 228 mm × 228 mm, 32 DWIs (b-values = 1000s/mm^2^) with diffusion gradients along different directions, and one non-DWIs (b-value = 0). An acquisition matrix of 112 × 112 was interpolated to 224 × 224, and 60 consecutive slices were acquired over 6 minutes. Raw data were exported to DTIStudio (Johns Hopkins University; https://www.mristudio.org/). An affine warp model in Automated Image Registration (AIR) tool was used to correct image distortions caused by eddy currents and misregistration errors due to head motion. After co-registration, rotational operations were extracted, and b-vector orientations were corrected. Tensors were estimated using linear least square fitting, and automatic outlier slice rejection was performed for relative error >3%. Maps of fractional anisotropy (FA), mean diffusivity (MD), AD, and RD were calculated.

### Measurements

**Fig 1:**
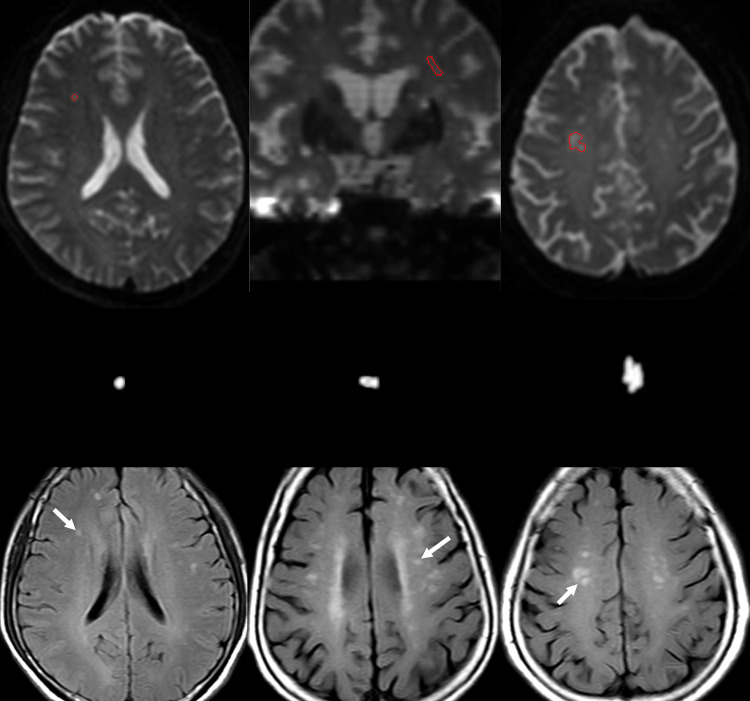
ROI determination of punctate, strip-shaped, and early fused lesions and the three-dimensional reconstruction after lesion segmentation. FLAIR images (bottom row) were used to differentiate WMH from perivascular spaces or lacunes.

The “magic wands” tool in DTIStudio [22] was used on B0 images to define 3D regions of interest (ROIs) for each lesion. Two experienced neuroradiologists (Yuan D and Min Z) used FLAIR sequences to distinguish WMHs from perivascular spaces or lacunes, resolving disagreements through consensus. Lesion locations were recorded based on colored FA map, and their direction of the maximum eigenvector (x, y and z representing left-right, anterior-posterior, and cephalo-caudal directions, respectively) were determined. Mean FA, MD, AD, and RD values were measured. ROIs were saved and converted to Analyze format using the ROIEditor. Lesions volume, major radius, and elongation were measured using Fiji’s 3D suite [23]. Since the morphology of lesions in 3D may differ from that in 2D, we used the elongation value to reclassify the extracted lesions. WMHs were classified as strip-shaped if elongation was ≥ 2 and punctate if < 2. Early confluent WMHs were irregularly shaped lesions not classified as punctate or strip-shaped. Elongation was defined as the ratio of the major to the second radius [24]. ROI determination and 3D reconstruction of lesions are shown in Fig 1. Mango software [25] (Lancaster, Martinez; www.ric.uthscsa.edu/mango) was used to determine the morphological principal axes direction (x, y or z) of each lesion. Consistency between the principal axis and the maximum eigenvector was defined as the alignment of the lesion’s principal axis with the direction of the maximum eigenvector (e.g., both oriented along the X-axis). This consistency was assessed visually by overlaying the eigenvector direction, derived from DTIStudio, onto the three-dimensional geometry of the lesion. Fiber Assignment by Continuous Tracking (FACT) algorithm was used to display fiber bundles [22], with ROIs placed at both ends of the principal axes of the strip-shaped lesions to demonstrate fiber bundles coursing along these axes.

### Statistical analysis

Statistical analyses were performed using SPSS (version 22, IBM, Armonk, NY, USA), with two-sided p-values <0.05 considered significant. Chi-square tests compared lesion distribution differences among three groups. We employed a mixed-effects model with random effects for subjects, followed by post hoc Bonferroni tests, to compare measurements of FA, MD, AD, and RD across groups. The p-values were adjusted for multiple comparisons. Age and lesion volume were included as covariates to control for their potential influence. The number of lesions with consistent principal axes and fiber directions was summarized in a cross table, and ratio differences were tested using chi-square.

## Results

A total of 996 WMH lesions were measured, including 739 punctate, 181 strip-shaped, and 76 early confluent lesions. The average age of the three groups were 65.0 ± 10.9, 66.2 ± 11.5 and 66.2 ± 12.2 years, respectively, with no significant differences in age or sex distribution(Table 1).

**Table 1:** Count(%) of distribution of punctate lesions (Elongation <2), strip-shaped lesions (Elongation ≥2), and early confluent lesions in the white matter.

	Punctate	Strip-shaped	Early confluent
CR	202 (27.3%)	43 (23.8%)	22 (28.9%)
SLF	183 (24.8%)	41 (22.7%)	22 (28.9%)
AFas	56 (7.6%)	14 (7.7%)	5 (6.6%)
CG	9 (1.2%)	2 (1.1%)	0 (0.0%)
AFib	190 (25.7%)	52 (28.7%)	16 (21.1%)
CC	54 (7.3%)	11 (6.1%)	7 (9.2%)
IFO	4 (0.5%)	0 (0.0%)	0 (0.0%)
OR	10 (1.4%)	9 (5.0%)	1 (1.3%)
IC	2 (0.3%)	1 (0.6%)	0 (0.0%)
MF	18 (2.4%)	5 (2.8%)	2 (2.6%)
EC	11 (1.5%)	3 (1.7%)	1 (1.3%)
Total	739	181	76
*X* ^*2*^	16.554		
*P-Value*	0.682		

Lesions spanning two white matter tracts were recorded in larger distribution areas. CR: corona radiata; SLF superior longitudinal fasciculus; AFas: arcuate fasciculus; CG cingulum; AFib: arcuate fiber; CC: corpus callosum; IFO: inferior occipitofrontal fasciculi; OR: optic radiation; IC: internal capsule; MF: fiber-minor forceps; EC: external capsule.

**Fig 2:**
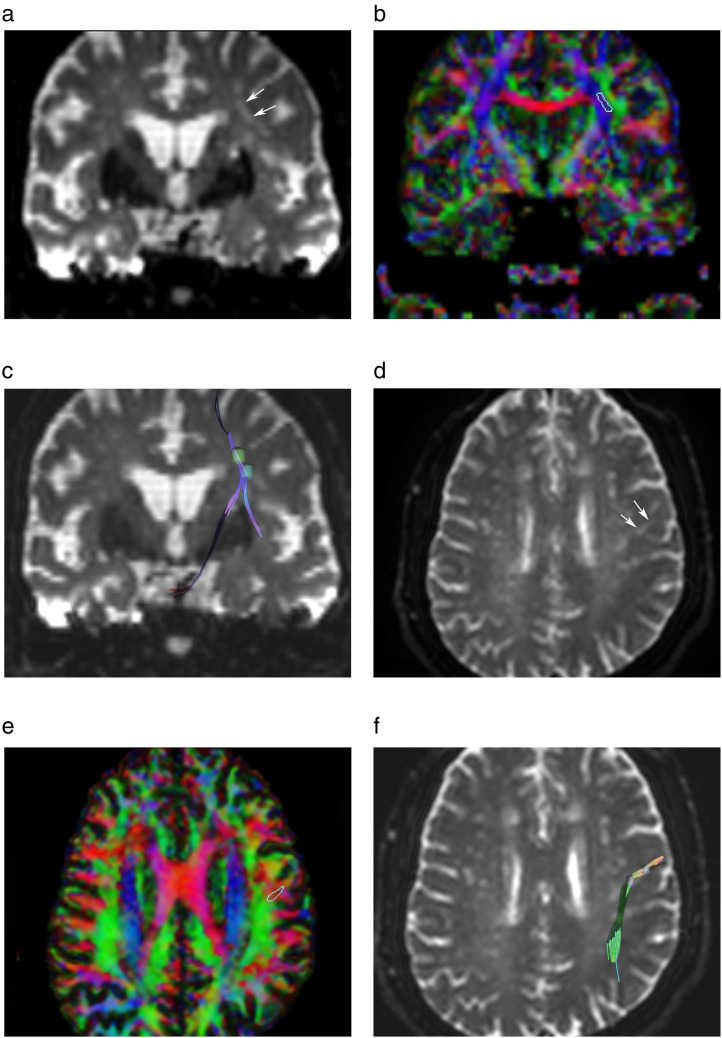
A strip-shaped WMH is located in the left corona radiata (a and b), with the principal axes (cephalo-caudal direction) aligned with the maximum eigenvalue direction (b). Fiber tracing demonstrates that the fiber direction follows the principal axes of the lesion (c). Another strip-shaped WMH is located within the arcuate fibers of the left frontal cortex (d and e), with the principal axes (left-right direction) aligned with the maximum eigenvalue direction (e). Fiber tracing confirms that the fiber direction aligns with the principal axes of the lesion (f).

Among the 181 strip-shaped WMHs, the principal axes of 104 lesions (57.5%) were consistent with their maximum eigenvector. Fiber tracking demonstrated fibers passing along the principal axes of WMHs (Fig 2). The proportion of consistent strip-shaped WMHs (57.5%) was significantly higher than that of punctate WMHs (44.7%) and early confluent WMHs (42.1%) (P < 0.05) (Table 2).

**Table 2:** Crosstab of WMH type and consistent direction.

WMH Type	Direction Consistent		Total
	Inconsistent (%)	Consistent (%)	
Punctate	409 (55.3)	330 (44.7)	739
Strip-shaped	77 (42.5)	104 (57.5)	181
Early confluent	44 (57.9)	32 (42.1)	76
Total	530	446	996

**Fig 3:**
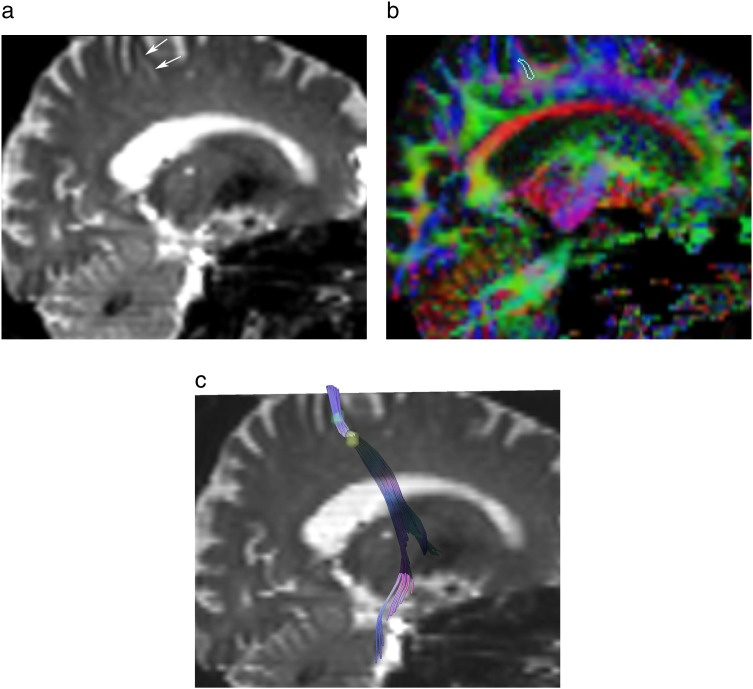
A strip-shaped WMH is located in the arcuate fibers of the left frontal lobe (a and b), with its principal axes (cephalo-caudal direction) not aligned with the maximum eigenvalue direction (anterior-posterior direction) (b). Fiber tracing shows that the fiber direction follows the principal axes of the lesion (c).

**Fig 4:**
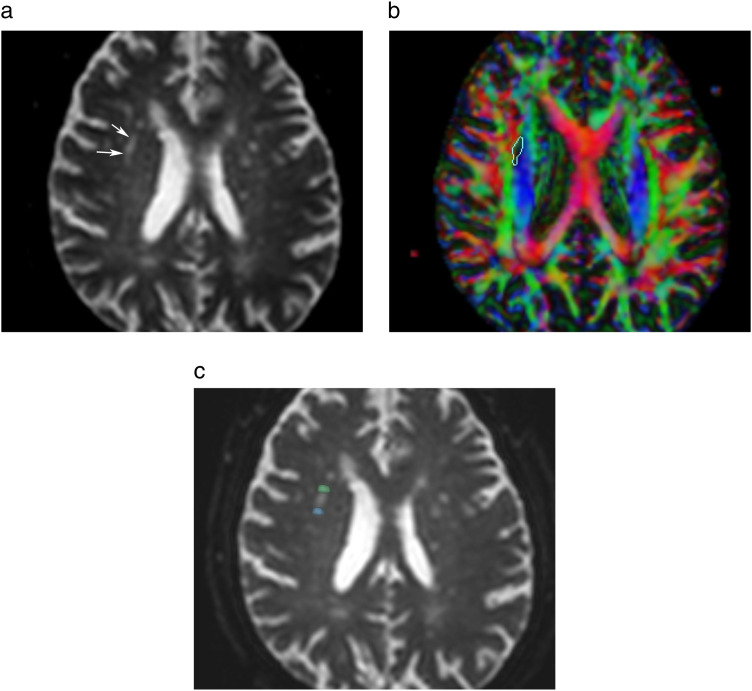
A strip-shaped WMH is principal located in the right corona radiata (a and b), with its principal axes (anterior-posterior direction) not aligned with the maximum eigenvalue direction (cephalo-caudal direction) (b). Fiber tracing reveals that the fiber direction does not align with the principal axes of the lesion (c).

Fiber tracing on 77 strip-shaped lesions with inconsistent principal axes and maximum eigenvalue directions resulted that 42 (54.5%) had fiber bundles passing along their principal axes (Fig 3), while 35 (45.5%) did not (Fig 4).

Significant differences were observed in volume, major radius, FA, MD, AD, and RD among groups (Table 3). Strip-shaped WMHs had higher volumes and major radius than punctate WMHs but lower values than early confluent WMHs (P < 0.001). Mixed-effects models revealed significant main effects of lesion type on all four DTI metrics: FA (F = 7.003, P < 0.001), MD (F = 8.419, P < 0.001), AD (F = 4.437, P = 0.012), and RD (F = 8.935, P < 0.001). Bonferroni-corrected post-hoc pairwise comparisons showed that the stripe group exhibited significantly higher FA values (M = 0.525, 95% CI [0.502, 0.549]) than the confluence group (M = 0.450, 95% CI[0.421, 0.478]), with a mean difference of 0.076 (P < 0.001, 95% CI[0.032, 0.119]). For MD, the stripe group showed significantly lower values (M = 5.643, 95% CI [5.508, 5.777]) than the confluence group (M = 5.931, 95% CI[5.769, 6.094]; MD=−0.288, P = 0.011, 95% CI [−0.525, −0.052]), but did not differ significantly from the punctate group (M = 5.630, 95% CI [5.497, 5.764]; MD = 0.013, P = 1.000, 95% CI[−0.183, 0.209]). For RD, the stripe group similarly exhibited significantly lower values (M = 3.893, 95%CI [3.729, 4.057]) than the confluence group (M = 4.440, 95% CI [4.241, 4.638]; MD = −0.547, P < 0.001, 95% CI[−0.842, −0.252]), but showed no significant difference from the punctate group (M = 4.060, 95% CI[3.898, 4.222]; MD = 0.167, P = 0.306, 95%CI [−0.078, 0.413]). Bonferroni-corrected pairwise comparisons revealed no significant differences in AD among the three groups (all P > 0.05). The random effect of participant was not significant for any of the four metrics (P = 0.430, 0.122, 0.671, and 0.112, respectively).

**Table 3:** Compare measurements adjusted with age and lesion volume among punctate lesions (Elongation<2), strip-shaped lesions (Elongation≥2), and early confluent lesions.

	Lesion Type	N	Mean	Standard error	95% confidence interval	F-Value	P-Value
FA	Punctate	739	0.490	0.012	0.467, 0.512	7.003	<0.001
	Strip-shaped	181	0.525	0.012	0.502, 0.549		
	Early confluent	76	0.450	0.015	0.421, 0.478		
MD (10^–4^mm^2^s^-1^)	Punctate	739	5.630	0.068	5.497, 5.764	8.419	<0.001
	Strip-shaped	181	5.643	0.069	5.508, 5.777		
	Early confluent	76	5.931	0.083	5.769, 6.094		
AD (10^–4^mm^2^s^-1^)	Punctate	739	8.778	0.126	8.531, 9.025	4.437	0.012
	Strip-shaped	181	9.131	0.131	8.873, 9.389		
	Early confluent	76	8.972	0.158	8.661, 9.283		
RD (10^–4^mm^2^s^-1^)	Punctate	739	4.060	0.082	3.898, 4.222	8.935	<0.001
	Strip-shaped	181	3.893	0.084	3.729,4.057		
	Early confluent	76	4.440	0.101	4.241, 4.638		

## Discussion

Our findings indicate 57.5% of strip-shaped WMHs have principal axes consistent with the direction of white matter fibers, a proportion significantly higher than that of punctate and early confluent WMHs. Additionally, significant differences in diffusion tensor metrics suggest that strip-shaped WMHs have distinct pathological features compared to punctate and early confluent WMHs. Fiber tracing revealed that some strip-shaped WMHs with inconsistent principal axes and maximum diffusivity directions still had fibers following their principal axes, possibly due to crossed fiber bundles masking the maximum eigenvalue direction [26]. The alignment of principal axes with fiber progression in most strip-shaped WMHs suggests a neurodegenerative origin, a spatial relationship not expected for randomly distributed perivascular or interstitial pathologies [17,18].

Strip-shaped WMH exhibited lower RD than early confluent WMHs. Although not statistically significant, there was a trend toward higher AD in the stripe-shaped group compared to the punctate group. The increased AD in strip-shaped WMHs contrasts with the decreased AD observed in early Wallerian degeneration [27], suggesting chronic axonal injury secondary to axons or astrocytic process loss. Similar changes have been observed in neurodegenerative diseases such as HIV [28], ALS [29], and Alzheimer’s disease [29]. The higher RD in early confluent WMHs is consistent with severe demyelination [30,31], supporting previous findings [9]. However, the lack of significant RD differences between strip-shaped and punctate WMHs suggest distinct pathological mechanisms. Of course, further pathological studies are paramount to corroborate this inference. It should also be noted that alterations in AD and RD could be confounded by shifts in water content, and their definitive relationship with neurodegeneration versus demyelination has yet to be fully established [31]. We employed a mixed-effects model incorporating participants as random effects, indicating that the findings from our lesion-level analysis were not driven by subject-level variability.

This study has several limitations. First, the use of an elongation threshold of 2 to distinguish punctate from strip-shaped WMH, while reducing subjective, is based solely on our experience. The current classification approach may have diminished the statistical significance of the observed differences, reflecting the underlying pathological complexity of white matter hyperintensities. Thus, developing more effective classification methods may require the inclusion of factors beyond elongation thresholds. Second, manual WMH segmentation is labor-intensive and potentially subjective, though the “magic wands” tool minimized operator influence. Automated segmentation methods were not used because they often fail to adequately segment peripheral deep WMHs, particularly in cases with low WMH burden [14]. Third, the use of B0 images for segmentation likely resulted in suboptimal contrast resolution for white matter hyperintensities (WMH), which contributed to the failure of our automated segmentation attempt. Although 3D FLAIR sequences provide superior lesion conspicuity and are better suited for segmentation, this sequence was not part of our imaging protocol. Incorporating such sequences represents an important direction for future methodological improvement. Fourth, no pathological confirmation or definitive imaging reference standard for Wallerian degeneration was available in this study. Consequently, the interpretation of strip-shaped WMHs as markers of chronic neurodegeneration remains inferential and should be viewed with appropriate caution.

## Conclusions

Using DTI, we demonstrated that strip-shaped WMHs exhibit distinct characteristics compared to punctate and early confluent WMHs, suggesting potential association with chronic axonal injury due to neurodegeneration.
